# “Song of Life”: Results of a multicenter randomized trial on the effects of biographical music therapy in palliative care

**DOI:** 10.1177/02692163211010394

**Published:** 2021-04-20

**Authors:** Marco Warth, Friederike Koehler, Martin Brehmen, Martin Weber, Hubert J Bardenheuer, Beate Ditzen, Jens Kessler

**Affiliations:** 1 Institute of Medical Psychology, Center for Psychosocial Medicine, University Hospital Heidelberg, Heidelberg, Germany; 2 Ruprecht-Karls-University Heidelberg, Heidelberg, Germany; 3 Interdisciplinary Palliative Care Unit, III. Department of Medicine, University Medical Center of the Johannes Gutenberg University of Mainz, Mainz, Germany; 4 Center of Pain Therapy and Palliative Care Medicine, Department of Anesthesiology, University Hospital Heidelberg, Heidelberg, Germany

**Keywords:** Music therapy, palliative care, cancer, randomized controlled trial, end-of-life, quality of life, spiritual well-being, distress, ego-integrity

## Abstract

**Background:**

Awareness for the importance of psychological and spiritual needs in patients with terminal diseases has increased in recent years, but randomized trials on the effects of psychosocial interventions are still rare.

**Aim:**

To investigate the efficacy of the “Song of Life” music therapy intervention regarding the emotional and psycho-spiritual dimensions of quality of life.

**Design:**

Patients were randomly assigned to either “Song of Life” or a relaxation intervention. “Song of Life” is a novel three-session music therapy intervention working with a biographically meaningful song. Primary outcome was the improvement in psychological quality of life. Secondary outcomes included spiritual well-being, ego-integrity, momentary distress, and global quality of life and the explorative assessment of treatment satisfaction (patient and family member version). Intention-to-treat analysis was conducted including adjustment for multiple testing in secondary outcomes.

**Setting/participants:**

Between December 2018 and August 2020, 104 patients receiving specialized palliative care were recruited from two palliative care wards.

**Results:**

No significant differences were found regarding psychological and global quality of life, but “Song of Life” participants reported significantly higher spiritual well-being (*p* = 0.04) and ego-integrity (*p* < 0.01), as well as lower distress (*p* = 0.05) than patients in the control group. Both patients’ and family members’ treatment satisfaction was higher after “Song of Life” with large between-group effect sizes on items asking for meaningfulness (*d* = 0.96) and importance (*d* = 1.00).

**Conclusions:**

Our findings provide evidence that “Song of Life” is an effective and meaningful biographical music therapy intervention to facilitate psycho-spiritual integration in terminally ill patients.

**Trial Registration:**

German Clinical Trials Register (DRKS)—DRKS00015308 (date of registration: September 7th 2018).

**What is already known about the topic:**

- Patients nearing the end of life report high needs for emotional and spiritual support.
- Although the use of psychosocial interventions including life review and creative arts-based techniques is immensely valued in clinical practice, recent guidelines, reviews, and reports have repeatedly called for high-quality studies on their efficacy.

**What this paper adds:**

- Findings show that biographical music therapy can effectively facilitate psycho-spiritual integration of past life events.
- The intervention was further effective in reducing patients’ momentary distress and was perceived as meaningful and important by both patients and family members.

**Implications for practice, theory or policy:**

- Together with previous findings, music therapy has been shown to be effective with regard to a number of clinically relevant outcomes including pain, quality of life, and spiritual well-being.
- The present study therefore marks an important step towards an evidence-based rationale for the use of music therapy in palliative care.
- The “Song of Life” intervention should be recommended to address emotional and existential needs in terminally ill patients nearing the end of life.

## Background

The diagnosis and course of a terminal disease is one of the most distressing life events with a detrimental impact on the physical, emotional, spiritual, and social well-being of the individual and their relatives.^1^ While medical interventions effectively treat physical symptoms like pain,^2^ the mission of palliative care is to provide holistic support of patients on all levels of well-being.^3^ For instance, one third of palliative care patients suffer from depression, adjustment, or anxiety disorders.^4^ Therefore, psychosocial interventions have emerged to specifically address emotional difficulties and spiritual concerns regarding meaning in life,^5^ including cognitive-behavioral therapy, mindfulness, life review or meaning-centered interventions, and creative arts-based therapies.^6–8^ Research on these treatments provides evidence for improvements in depression and quality of life, particularly in advanced cancer patients.^9–12^ However, the majority of these interventions were designed for patients in non-final stages of the disease requiring a session number that is hard to achieve in patients nearing the end of life. Palliative care settings often deal with unique conditions necessitating respective adjustments in intervention protocols.^13^

A recent meta-analysis summarized studies using brief interventions with four sessions or less showing improvements in quality of life and reductions in emotional and existential distress.^14^ Most of these studies focused on life review techniques or music therapy. The common therapeutic focus of life review interventions (e.g. dignity therapy^15–17^) lies on the patient’s biography and the attempt to create a legacy,^7,18^ as generativity and ego-integrity (i.e. achieving a sense of meaning and acceptance of past life events) were postulated as important developmental tasks at the end of life.^19,20^ While a recent meta-analysis provided evidence for improvements in psychological well-being, efficacy trials revealed inconsistent findings concerning quality of life and dignity.^21^

Creative arts therapies offer an alternative way for terminally ill patients to regulate emotions and integrate life experiences on a psycho-spiritual level.^22^ Music therapy has been a substantial part of palliative care since its beginnings^23^ and aims at the improvement of quality of life by alleviating physical and emotional burden as well as through enabling communication and spiritual experiences.^24^ Common definitions of music therapy emphasize the importance of the therapeutic relationship combined with musical and verbal techniques, and thereby contrast music listening interventions which do not require the presence of a trained therapist.^25^ Music therapy techniques encompass receptive (e.g. music and imagery), creative (e.g. songwriting), recreative (e.g. instrument playing), and combined (e.g. musical life review) methods,^26^ which usually are customized to the individual’s needs.^27^ Previous trials on the efficacy of music therapy in palliative care reported beneficial effects regarding the improvement of quality of life, general well-being,^28,29^ and spiritual well-being,^30,31^ as well as the reduction of pain^32^ and anxiety.^33^ No study has systematically evaluated biographical music therapy in a clinical trial in palliative care yet.

Therefore, the aim of the present study was to investigate the efficacy of the newly developed, pilot-tested^34^ biographical music therapy technique “Song of Life” (SOL) with palliative care patients. Working with a biographically meaningful song, SOL integrates elements of life review interventions and creative arts therapies. Hence, we hypothesized the SOL to have more beneficial effects on the psychological and spiritual domain of quality of life than a non-specific psychosocial control treatment.

## Methods

### Study design

This multicenter randomized controlled trial compared the effects of the SOL music therapy intervention plus usual care as the experimental group with a relaxation intervention (RELAX) plus usual care as the control group in a parallel design. Patients in both groups participated in three sessions of 20–30 min duration. We additionally recruited family members for participation in an assessment of treatment satisfaction. The trial was preregistered at the German Clinical Trials Registry (DRKS00015308) and received approval by the local ethics committees. Methods and procedures have been published in a study protocol.^35^

### Setting

Study sites were the University Palliative Care Unit at St. Vincentius Hospital, Heidelberg University Hospital, Germany, and the Interdisciplinary Palliative Care Unit at the University Medical Center of the Johannes Gutenberg University of Mainz, Germany.

### Randomization, masking, and blinding

Randomization to one of the two study arms was based on a computer-based block randomization sequence (block size = 8) stratified by study site, which was created by a researcher not involved in recruitment, assessment, or treatment. Sequentially numbered opaque envelopes were opened after the patient had provided informed consent and completed baseline assessment. Adhering to recommendations regarding blinding of participants, we (a) used an active control group without the patient knowing which one was the experimental group and (b) implemented an expectancy measure at baseline,^36^ assessed via the item “I expect the study intervention to be helpful to me” (agreement, 1–5). While patients were blind to the study hypothesis, blinding of therapists and outcome assessors was not feasible.

### Participants

Patients from these palliative care wards were included if they (a) received palliative treatment according to OPS 8-982/OPS 8-98e (German modification of International Classification of Procedures in Medicine; ICPM) or had an estimated life expectancy of <12 months,^37^ (b) were ⩾18 years old, and (c) were able to provide informed consent. Patients were excluded if they (d) did not speak German language, (e) had a clinical estimation of life expectancy <1 week, or (e) showed cognitive or auditory impairments, or psychiatric symptoms. Patients were asked to name a family member or another close person to be included in an evaluation of treatment satisfaction. Family members were included if they (a) were ⩾18 years old and (b) spoke German language.

### Sampling and recruitment

**Figure 1.**
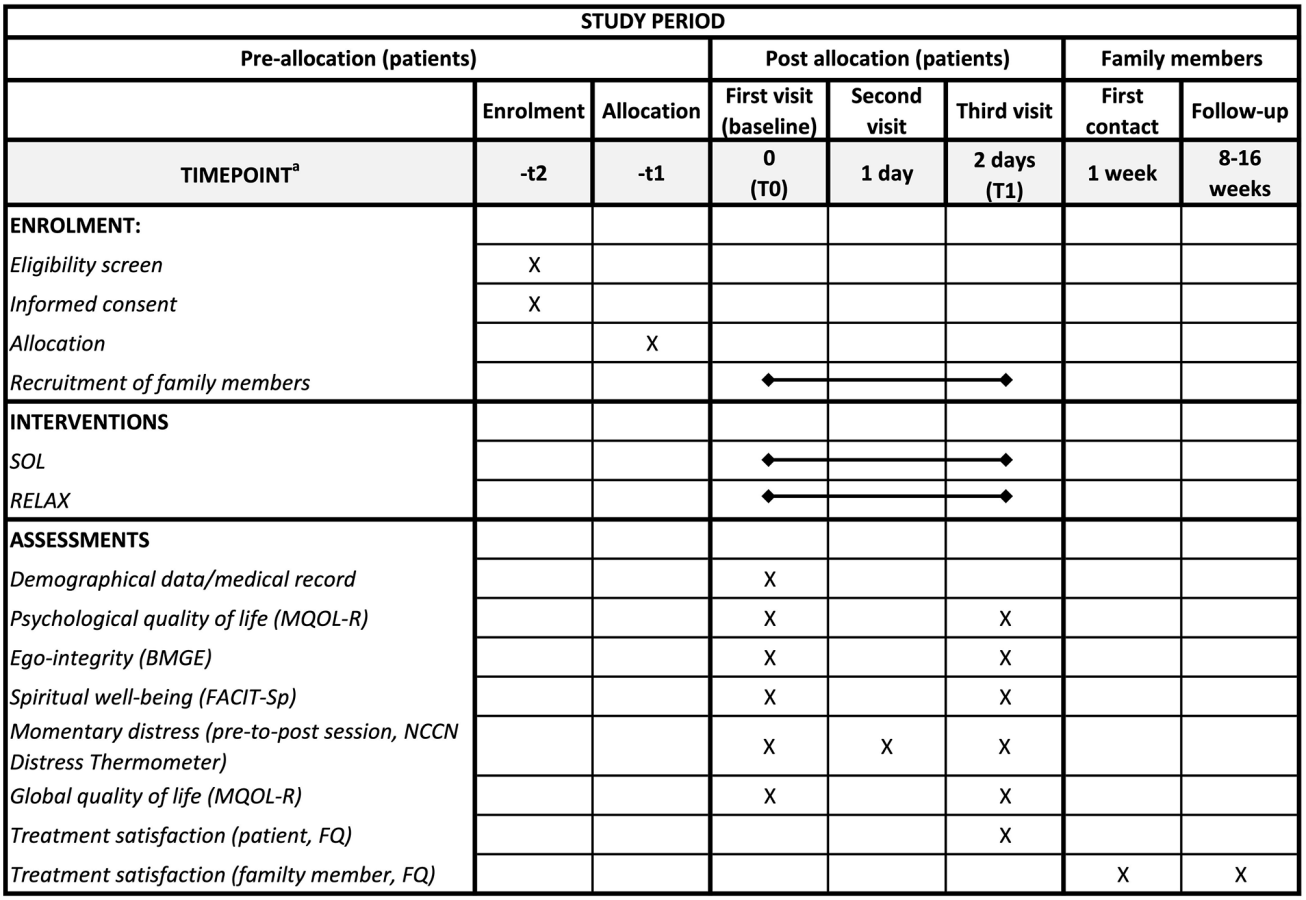
Figure 2 SPIRIT figure: schedule of enrolment, interventions and assessments. SPIRIT: standard protocol items: recommendations for interventional trials; SOL: “Song of Life” music therapy; RELAX: relaxation intervention; MQOL-R: McGill quality of life questionnaire–revised); BMGE: brief measure of generativity and ego-integrity; FACIT-Sp: functional assessment of chronic illness therapy-spiritual well-being; NCCN: national comprehensive cancer network; FQ: feedback questionnaire. ^a^Deviations from the schedule were possible.

Based on the medical record and information from the physician, eligible patients from the two palliative care units were contacted at bedside consecutively between December 2018 and August 2020. All patients were informed about the study goals, procedures, risks, and benefits and were asked to provide written informed consent. Family members were contacted after allocation of the patient to one of the interventions and were also asked to provide informed consent before inclusion in the study (Figure 1).

### Interventions

The SOL intervention was a new pilot-tested^34^ music therapy technique, in which therapist and patient explored and identified a biographically meaningful and emotionally arousing song in the first session. This song was played live to the patient in the second session by the therapist in a lullaby style (i.e. 3/4 or 6/8 rhythm, slow tempo) with guitar or e-piano and voice, while the session was audio-recorded. A CD or flash drive containing the edited recording was handed over to the patient as a possible legacy in the third session. Both patient and therapist listened to the recording and reflected on feelings and memories, guided by pre-defined questions. Conversations in the last session were again audio-recorded.

Sessions in the control group consisted of three standardized relaxation interventions focusing on (a) muscle relaxation, (b) breathing, and (c) imaginary journey, plus a brief inquiry at the end of each session. Relaxation and mindfulness exercises were chosen as they proved to be feasible, safe, and potentially effective in seriously ill patients,^10^ but were non-specific with regard to psycho-spiritual integration processes targeted in the SOL intervention. Hence, exercises did not contain any biographical, spiritual, or musical content, and were delivered in person by the study therapists. Detailed intervention manuals are presented in Appendix A.

In both groups, sessions were preferably carried out on three consecutive days, but deviations from this schedule were possible (e.g. for organizational reasons or if wished by the patient). The two study therapists were trained music therapists with a wide range of experience and were employed at the two participating palliative care wards. The study therapists participated in a 36-h training program conveying all intervention procedures. Moreover, study therapists were asked to rate their perception of adherence to the intervention protocol for each patient and of the musical realization of the SOL (e.g. using rhythm, tempo, and dynamics in accordance with the training and manual; see Appendix A) on a scale from 1 (“not at all”) to 5 (“very good”). Moreover, audio recordings in the experimental group (sessions 2 and 3) were rated by a research assistant for protocol adherence (e.g. interview topics covered; see Appendix A) on a self-developed treatment integrity scale (1–5). Further, study therapists received supervision by the principal investigator after 25%, 50%, and 75% of recruitment completion to maintain high treatment fidelity, where therapists were asked to describe cases, treatment progress, or difficulties and received feedback by the supervisor.

### Data collection

After obtaining written informed consent, research staff initiated baseline assessment (T0) of outcome measures listed below and afterwards opened an envelope with the group assignment. Each of the three intervention sessions contained a pre-to-post single-item assessment of momentary distress. Post-intervention outcome data (T1) was immediately assessed after session 3.

Family members who provided informed consent completed a brief evaluation of treatment satisfaction after the intervention (T1) and again after 8–16 weeks (T2) either in person or by phone. The rationale of these follow-up assessments was to gather indirect information on the endurance of treatment effects, as previous trials showed that time spans for patient outcome assessments of more than 1 week were not feasible in palliative care research.^31^ Figure 1 gives an overview of the assessment procedures.

### Outcome measures

The working mechanism of the SOL intervention was assumed to include the emotional and spiritual processing and integration of past life events. Outcomes have been chosen in accordance with this assumption and a detailed rationale for the selection of study outcomes can be found in the study protocol.^35^

The primary study outcome was the pre-to-post change (T0-T1) in *psychological quality of life* (range: 0–10) as measured by the psychological subscale of the validated McGill Quality of Life Questionnaire – Revised (MQOL-R),^38^ which contains four items asking for anxiety and depression in the past 2 days.

Secondary outcomes included the 5-item *ego-integrity* subscale (1–5) of the validated Brief Measure of Generativity and Ego-Integrity^20^ (BMGE) for the assessment of acceptance and the sense of meaning regarding one’s past life. Changes in non-religious aspects of *spiritual well-being* were measured by the validated 8-item meaning/peace-scale (0–32) of the Functional Assessment of Chronic Illness Therapy – Spiritual Well-Being (FACIT-Sp), which is commonly used in palliative care research.^39,40^ Due to the intervention duration of 3 days, the item time frame was reduced from 7 to 3 days. Moreover, we assessed patient’s momentary *distress* before and after each session by use of the modified version of the validated single-item (0–10) NCCN Distress Thermometer asking for acute distress.^41,42^ For consistency with other outcome measures, we analyzed the two distress scores before the first session and after the last. The MQOL-R’s single item (0–10) on *global quality of life* during the past 2 days additionally served as a secondary endpoint.^38^

Outcome assessment was complemented by modified versions of the Feedback Questionnaire (FQ), in the patient^16^ and family member^43^ version. The FQ is a non-validated retrospective measure frequently used in research on biographical interventions in palliative care. Both versions used eight items (agreement from 1 to 5) to cover the *perception of treatment satisfaction* (T1: patients and family members, T2: family members only). All questionnaires were delivered by research assistants who were not involved in the treatment process. The timing of assessments is displayed in Figure 1.

### Statistical analyses

A priori calculations with G*Power^44^ assuming a medium-sized effect of Cohen’s *d* = 0.50^45^ on the primary outcome, statistical power of (1–ß) = 0.8, a type-I error probability of α = 0.05, a correlation between covariate and outcome of ρ = 0.6, and an attrition rate of 25% revealed an optimal sample size of *N* = 104 patients for this study. The rationale for the estimation of parameters has been discussed in the study protocol.^35^

Hypotheses were tested by intention to treat^46^ in the statistical environment R.^47^ We first calculated analyses of covariance (ANCOVA) with a multiply imputed dataset (MI), using the baseline score as a covariate, treatment and study site^48^ as fixed factors, and the post-intervention score as the outcome. We created 20 imputations and combined the statistical results using the *R* package “mice.”^49^ Effect sizes (Cohen’s *d*) and 95% confidence intervals (CI) were computed according to Morris’^50^ formula for pretest-posttest-control-group designs and were visualized with the funnel plot function in the “metaphor”^51^ package. Next, sensitivity analysis with all available data (AAD) was calculated by multilevel modeling using the “lme4” package.^52^ We chose to conduct analyses with both ANCOVA (MI) and multilevel modeling (AAD) as different approaches to handle missing data and expected similar results. Parameters were obtained via restricted maximum likelihood (REML) estimation. We computed a random-intercept model including the pre- and post-intervention scores as the outcome, and time, treatment, time × treatment, and study site as fixed factors. Concerning the analyses of secondary outcomes, we accounted for the false discovery rate (FDR) in multiple comparisons employing the Benjamini-Hochberg correction and report adjusted *p*-values.^53^ Both α and the FDR were set on 0.05. Group differences on the FQ were exploratively analyzed on an item level^16^ using Cohen’s *d* and 95 % CIs calculated with the “effectsize”^54^ package, as this non-validated measure does not allow for calculation of a composite score.

## Results

**Figure 2.**
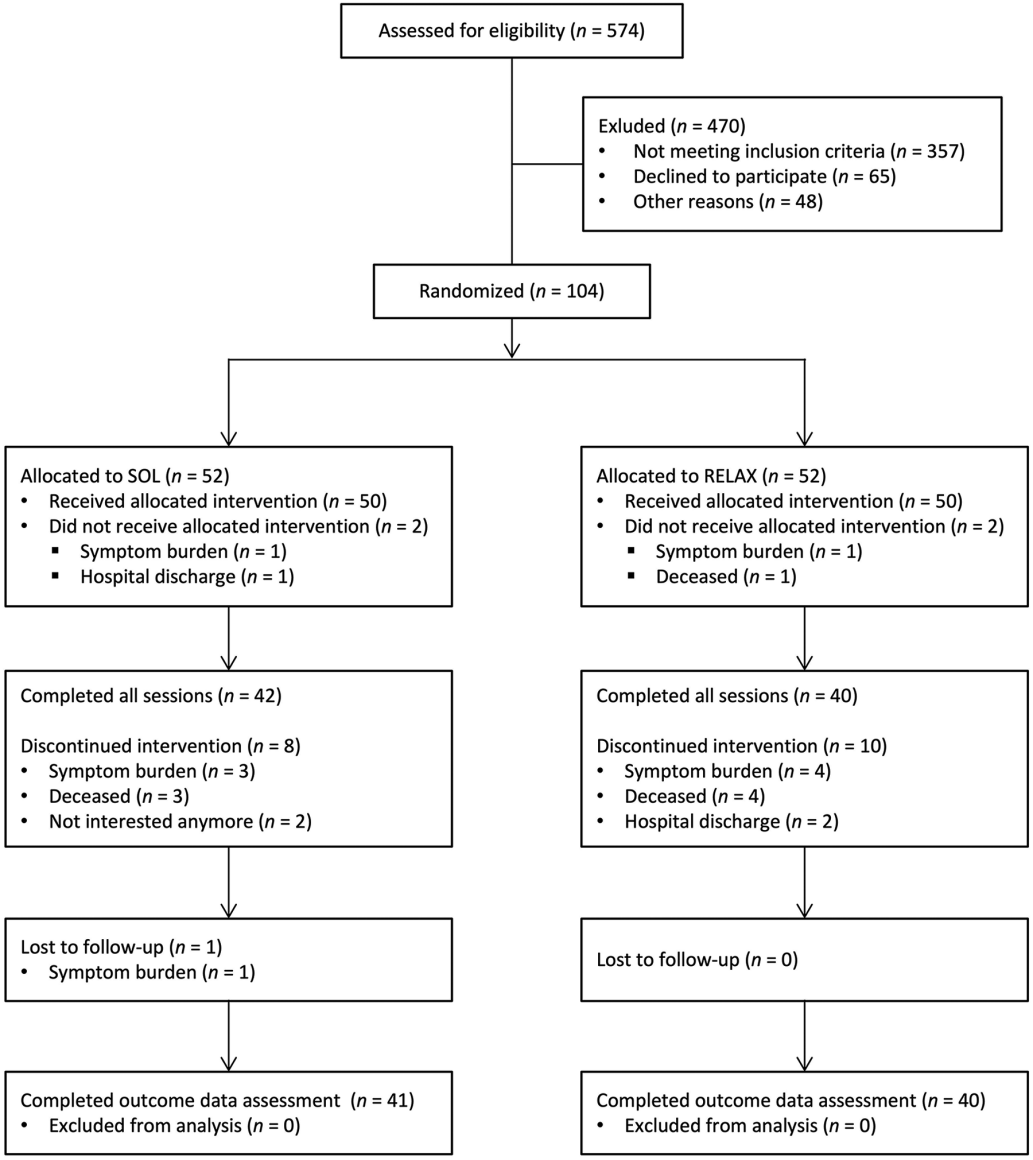
Patient flow chart. SOL: “Song of Life” music therapy; RELAX: relaxation intervention.

Of the 574 patients assessed for eligibility between December 2018 and August 2020, *N* = 104 consented to participate and were randomly assigned to either SOL (*n* = 52) or RELAX (*n* = 52). Among those included, *n* = 100 patients (98.0%) completed session 1, *n* = 89 completed session 2 (85.6%), and *n* = 82 (78.8%) completed the entire intervention protocol. Eighty-one patients (77.9%) provided complete outcome data, hence, the attrition rate in the present study was 22.1%. The patient flow chart is shown in Figure 2.

The majority of patients was female (*n* = 77, 74.0%) and all but *n* = 2 patients were primarily diagnosed with advanced cancer (*n* = 102, 98.1%). The mean age was *M* = 66.1 years (SD = 12.0). As Table 1 shows, study groups were comparable with regard to age, sex, diagnosis, functional status, and treatment expectancy. On average, therapists rated their subjective overall adherence to the treatment protocols at 3.52 (SD = 0.82) in the experimental group and 3.74 (SD = 0.94) in the control group. In the experimental group, the musical adherence in session 2 received an average rating of *M* = 3.50 (SD = 0.90) by the therapists and of *M* = 4.47 (SD = 0.67) by the research assistant. Adherence to the interview guidelines in session 3 (SOL) was rated 3.36 (0.90) by the research assistant. The majority of chosen songs were associated with a meaningful life phase, close relationships, or important life event, while other songs represented the lifelong companionship and consolation through music in the patient’s biography.

**Table 1.** Baseline sample characteristics.

Characteristic	SOL	RELAX
Participants per site (*n*, %)		
Study site 1	24 (23.1%)	24 (23.1%)
Study site 2	28 (26.9%)	28 (26.9%)
Age (*M*, SD, years)	67.75 (11.5)	64.46 (12.37)
Sex (*n*, % female)	41 (78.8%)	36 (69.2%)
Cancer type (%, *n*)		
Gastrointestinal	30.8% (16)	26.9% (14)
Gynecologic	28.8% (15)	26.9% (14)
Skin	9.6% (5)	9.6% (5)
Lymphatic	5.8% (3)	9.6% (5)
Thoracic	9.6% (5)	15.4% (8)
Other	13.5% (7)	9.6% (5)
Non-cancer	1.9% (1)	1.9% (1)
Karnofsky performance status scale (*M*, SD, 0–100)	42.88 (15.51)	48.46 (20.71)
Treatment expectancy (*M*, SD, 1–5)	3.74 (0.69)	3.81 (0.60)

SOL: “Song of Life” music therapy (experimental group); RELAX: relaxation intervention (control group); M: mean; SD: standard deviation.

**Figure 3.**
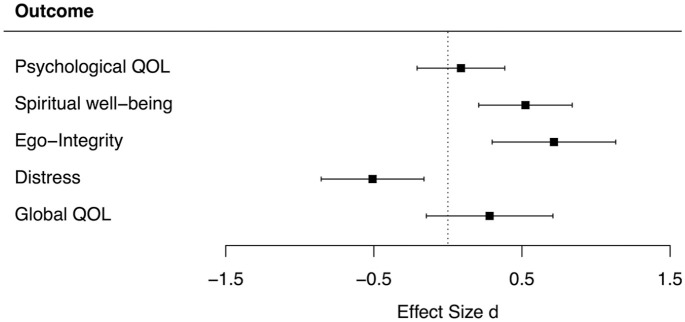
Effect sizes and 95% confidence intervals for primary and secondary outcomes. QOL: quality of life; *d*: effect size according to Morris;^50^ positive effect size indicates higher increase in SOL than in RELAX.

ANCOVA showed no statistically significant differences between groups in the primary outcome psychological quality of life (*F* = 0.13, *p* = 0.72, Table 2). With regard to secondary outcomes, patients reported significantly higher spiritual well-being (*F* = 5.53, *p* = 0.04) and ego-integrity (*F* = 16.03, *p* <* *0.01) after SOL than after RELAX. Effect sizes were medium to large for both spiritual well-being (*d* = 0.52) and ego-integrity (*d* = 0.72, Figure 3). Further, momentary distress was significantly lower after SOL (*F* = 4.49, *p* = 0.05), with a medium effect size of *d* = −0.51. Findings with regard to global quality of life showed a small between-group effect favoring the SOL (*d* = 0.28), but differences were not statistically significant (*F* = 0.71, *p* = 0.40). Sensitivity analyses using multilevel modeling with AAD yielded identical test results (Table 2).

**Table 2.** Results for primary and secondary outcomes.

	Descriptive statistics and effect sizes (AAD)							ANCOVA (MI)		MLM (AAD)	
	SOL			RELAX			*d* (CI)^a^				
	*N*	*M*	*SD*	*N*	*M*	*SD*		*F*	*p*^b^ Value	*b*	*p*^b^ Value
Psychological quality of life (MQOL-R, range = 0–10)											
Baseline (T0)	52	5.22	2.25	52	5.50	2.74					
Post-intervention (T1)	41	5.46	2.07	40	5.51	2.44	0.09 (−0.21, 0.38)	0.13	0.721	0.07	0.859
Meaning/peace (FACIT-Sp, range = 0–32)											
Baseline (T0)	50	20.40	4.45	50	22.70	4.32					
Post-intervention (T1)	41	22.32	5.09	40	22.30	5.17	0.52 (0.21, 0.84)	5.53	0.039*	2.45	0.006*
Ego-integrity (BMGE, range = 1–5)											
Baseline (T0)	51	3.20	0.52	52	3.34	0.66					
Post-intervention (T1)	41	3.58	0.57	40	3.29	0.59	0.72 (0.30, 1.13)	16.03	<0.001*	0.47	0.001*
Distress (NCCN distress thermometer, range = 0–10)											
Baseline (T0)	50	5.66	2.50	50	4.82	2.54					
Post-intervention (T1)	42	3.33	2.43	40	3.78	2.54	-0.51 (-0.86, -0.16)	4.49	0.046*	−1.18	0.017*
Global quality of life (MQOL-R, range = 0–10)											
Baseline (T0)	52	4.50	2.10	52	4.92	2.46					
Post-intervention (T1)	41	5.73	2.05	40	5.50	2.32	0.28 (-0.15, 0.71)	0.71	0.401	0.61	0.254

SOL: “Song of Life” music therapy; RELAX: relaxation intervention; ANCOVA: analysis of covariance; MLM: multilevel modeling; MI: multiple imputation; AAD: all available data; M: mean; SD: standard deviation; MQOL-R: McGill quality of life questionnaire–revise); FACIT-Sp: functional assessment of chronic illness therapy-spiritual well-Being; BMGE: brief measure of generativity and ego-integrity; NCCN: national comprehensive cancer network.

a Effect size variant of Cohen’s *d* for pretest-posttest-control group designs.^55^

b *p*-Values were adjusted by the Benjamini-Hochberg correction in secondary outcomes.^48^

* Statistically significant (*p* < 0.050).

Table 3 lists results of the explorative analyses for the FQ. Overall, patients and family members reported a positive impact of both interventions. Effect sizes between groups were medium to large or large, all favoring SOL over RELAX. In the patient version, large effect sizes were found on the items “Participating in the intervention made me feel that life was more meaningful” (*d* = 0.96) and “. . . was important to me” (*d* = 1.00). For family members, large between-group effect sizes were identified for the items “. . . made me feel something lasts from the patient” (*d* = 2.03) and “. . .will be helpful and comforting to our family” (*d* = 1.14), both immediately after the intervention (T1) and at follow-up (T2).

**Table 3.** Explorative results for feedback questionnaire.

Participating in the intervention…	Post-intervention (T1, AAD)					Follow-up (T2, AAD)				
	SOL		RELAX			SOL		RELAX		
Patient evaluation	*n*	*M* (SD)	*n*	*M* (SD)	*d* [95% CI]	*n*	*M* (SD)	*N*	*M* (SD)	*d* [95% CI]
. . . was helpful to me	40	4.30 (0.69)	40	3.80 (0.88)	0.63 [0.18, 1.08]		–		–	–
. . . was satisfactory	40	4.55 (0.71)	40	3.98 (0.80)	0.76 [0.30, 1.21]		–		–	–
. . . made me feel that life was more meaningful	40	3.60 (0.98)	40	2.58 (1.15)	0.96 [0.49, 1.42]		–		–	–
. . . met my expectations	36	4.22 (0.72)	39	3.64 (0.93)	0.69 [0.23, 1.16]		–		–	–
. . . was or will be of help to my family	39	3.56 (0.99)	39	2.69 (1.24)	0.78 [0.32, 1.24]		–		–	–
. . . helped me to accept the way things are	40	3.70 (0.79)	40	3.05 (1.11)	0.68 [0.22, 1.13]		–		–	–
. . . was important to me	40	4.35 (0.70)	40	3.53 (0.93)	1.00 [0.53, 1.47]		–		–	–
. . . is something I would recommend to others	40	4.70 (0.52)	39	4.33 (0.58)	0.67 [0.22, 1.12]		–		–	–
Family member evaluation										
. . . was helpful to the patient	23	4.52 (0.59)	19	3.58 (0.90)	1.26 [0.59, 1.93]	17	4.59 (0.51)	9	3.67 (0.87)	1.42 [0.52, 2.32]
. . . was satisfactory to the patient	23	4.61 (0.50)	19	3.63 (1.01)	1.26 [0.60, 1.93]	16	4.38 (0.50)	9	3.44 (1.13)	1.19 [0.31, 2.08]
. . . made the patient feel life was meaningful	23	3.43 (1.12)	20	2.60 (1.14)	0.74 [0.12, 1.36]	16	3.13 (1.02)	9	2.00 (1.00)	1.11 [0.23, 1.98]
. . . helped to alleviate the patient’s suffering	22	4.23 (0.75)	20	3.20 (0.89)	1.25 [0.58, 1.91]	17	4.12 (0.70)	9	3.22 (1.20)	1.00 [0.14, 1.85]
. . . was helpful to me	23	3.78 (1.04)	19	2.95 (1.31)	0.71 [0.09, 1.34]	17	3.76 (1.15)	9	2.44 (1.24)	1.12 [0.25, 1.99]
. . . will be helpful and comforting to our family	22	3.86 (1.04)	19	2.53 (1.31)	1.14 [0.48, 1.81]	17	3.82 (1.19)	9	2.00 (1.12)	1.57 [0.65, 2.49]
. . . made me feel something lasts from patient	23	3.91 (1.16)	19	1.89 (0.74)	2.03 [1.28, 2.78]	17	3.82 (1.07)	9	1.67 (0.87)	2.14 [1.13, 3.14]
. . . is something I would recommend to others	23	4.83 (0.39)	20	4.15 (0.88)	1.02 [0.39, 1.66]	17	4.88 (0.33)	9	4.22 (0.83)	1.20 [0.32, 2.07]

Range = 1–5 (agreement); SOL: “Song of Life” music therapy; RELAX: relaxation intervention; AAD: all available data; M: mean; SD: standard deviation; d: effect size Cohen’s *d*; CI: confidence interval.^56^

## Discussion

### Main findings/results of the study

Overall, our findings provide evidence that the innovative SOL technique has a positive impact on emotional and spiritual components of palliative care patients’ quality of life. In particular, we found significant treatment benefits with medium effect sizes regarding higher spiritual well-being and ego-integrity in the SOL group. Since patients nearing the end of life name existential fears regarding meaning in life as a fundamental challenge,^55^ these results suggest that SOL provides relief through facilitating the experience of a sense of connectedness with life and oneself. Corroborating research on other life review techniques, the present study adds to the promising potential of biographical interventions with terminally ill patients pointing to personal meaningful music as a valuable legacy. In addition, the significant reduction of distress through SOL music therapy hints at an acute relief of burden as well. Patients at the end of life commonly report a high level of psychological distress involving unpleasant emotional experiences, so even a short-term decrease might be of clinical importance to alleviate the patients’ suffering.^57,58^ Similar to other studies investigating psychosocial interventions with advanced cancer patients, we found no significant differences between the study groups with regard to more general outcomes.^16^ As our primary outcome contained items on general depression and anxiety, the measurement might not depict the actual content and goals of SOL (i.e. psycho-spiritual integration processes). Additionally, without a third study arm receiving usual care only, we cannot make conclusions about the effects of either SOL or RELAX on the MQOL-R domains in comparison to no add-on treatment. Further, as patient-reported outcomes may depend on the individual interpretation of items, future research may include external assessments, for example, by clinicians, as well. Regarding the explorative evaluation of treatment satisfaction (FQ), patients and family members in the SOL group rated the intervention more favorably on all items. Single-item effect sizes point to a profound personal relevance and perceived meaningfulness of the SOL. Family members, in particular, considered SOL benefits to be enduring and helpful to the whole family. The large between-groups effect sizes at both T1 and T2 on the item “. . . made me feel something lasts from the patient” highlights the important difference that the created legacy (SOL recording) might have made for the (bereaved) family member.

### Limitations

With regard to the selection of an appropriate comparator for the experimental intervention, the Declaration of Helsinki demands the effects of a new intervention to be tested against those of the best proven intervention.^59^ Usual-care-only groups may therefore be considered unethical in palliative care research and may also threaten the internal validity of a study through attention bias and lack of blinding.^36^ Therefore, we decided to compare two active psychosocial therapies which may partially explain the non-significant findings regarding psychological and global quality of life. Moreover, the generalizability of findings may be limited as 74% of participants were female. Ad-hoc analysis of sex differences in the observed treatment effects showed that effect sizes for the increase in spiritual well-being were larger in women while the observed distress reductions were more pronounced in men. This finding is consistent with previous research suggesting that women may be more open to development in gratitude and compassion, while men may have a stronger disease- or health-related focus.^58^ Further, the patient assessment only contained two measurement times, as long-term assessment plans were proven to be unfeasible considering an average hospital stay of less than 2 weeks.^31,60^ The present attrition rate of 22.1% is comparable to other studies and we addressed the issue of missing data integrating findings from different statistical approaches.^61^ We implemented the T2 family member follow-up to evaluate the endurance of effects, but also faced recruitment challenges and high attrition rates increasing the likelihood of selection bias in these results. At T2 among the family members that participated, only 28.6% of patients were alive. Of note, there was a great amount of family members who declined to participate at T2 due to the recent loss of their loved one.

### What this study adds

Findings from this multicenter RCT suggest that SOL music therapy can serve as an effective psychosocial treatment in palliative care to facilitate psycho-spiritual integration and reduce distress in patients nearing the end of life. Future studies may continue to explore optimal study outcomes and should address the importance of patient characteristics (e.g. sex, cultural background) in order to tailor biographical interventions to the individual’s situation and needs.

## Supplemental Material

sj-docx-1-pmj-10.1177_02692163211010394 – Supplemental material for “Song of Life”: Results of a multicenter randomized trial on the effects of biographical music therapy in palliative care Click here for additional data file. Supplemental material, sj-docx-1-pmj-10.1177_02692163211010394 for “Song of Life”: Results of a multicenter randomized trial on the effects of biographical music therapy in palliative care by Marco Warth, Friederike Koehler, Martin Brehmen, Martin Weber, Hubert J Bardenheuer, Beate Ditzen and Jens Kessler in Palliative Medicine
