# Supplementary material for: “Song of Life”: Results of a multicenter randomized trial on the effects of biographical music therapy in palliative care
Source: Palliat Med. 2021 Apr 20;35(6):1126–36. doi: 10.1177/02692163211010394 (PMC8188998; doi:10.1177/02692163211010394)
Supplement: sj-docx-1-pmj-10.1177_02692163211010394 – Supplemental material for “Song of Life”: Results of a multicenter randomized trial on the effects of biographical music therapy in palliative care [file sj-docx-1-pmj-10.1177_02692163211010394.docx]

**Appendix A: Intervention Manual for Experimental Group (“Song of Life”) and Control Group (Relaxation)**

1. **Experimental Group „Song of Life“ (3 sessions)**

***Session 1: Exploration***

*Note: The following statements and questions are exemplary and provide possibilities to systematically explore the “Song of Life”. You don’t need to ask all of the questions and you don’t need to strictly follow the order. The procedure won’t be the same for every patient. If the conversation shifts in other directions, allow it or use your own questions to deepen specific topics and set impulses.*

**Introduction:**

Music accompanies us through our whole life: Mothers sing lullabies for their infants, children’s songs play an important role in learning, music helps adolescents to develop their identity, and many meaningful life events in adulthood, such as marriage, are associated with specific music. First of all, I would like to know what role music has generally played in your life.

- What kind of music did/do you like to listen to? (e.g., in different phases of life)
- Did you play an instrument or did you sing?
- Were there any persons in your life who inspired you with regard to music?
- Do you relate music to specific persons in your life?

Now, I would like to support you in finding a song that has a very special meaning in your life. Often, there are songs which we immediately associate with important persons, events or places in our lives as they evoke particular memories in us. We don’t need to find the most important song of your life. It is completely enough to discover a song which elicits images and maybe pleasurable feelings in you. You cannot do anything wrong here. Don’t worry that the song might be too difficult or unknown; we can figure it out together afterwards. When you can think of several songs, please tell me all of them. When you can’t remember the title or interpret of the song, we can look it up together.

- Take some time now. Maybe you already have a spontaneous idea in which direction this might be heading. [short break]
- Maybe it could be helpful to remember important persons, events or places in your life. Are there any songs you associate with them? [short break]

**Specific questions (if necessary):**

- Sometimes songs may remind us of **specific persons**. For example, can you think of a song that your parents sang for you or a song that you sang for your own children? Or a song that you sang or heard with friends or in a choir?
- Often, we also associate songs with **specific situations or events**. This could for example be marriage, birth or baptism. Is there a song that immediately brings up images and memories when you listen to it?
- **Important places** in our life can also be connected with specific songs. When you think about meaningful places in your life, maybe your favorite place in childhood or an important journey, can you think of a song that shaped this place?
- Songs can also accompany **specific phases in our life**. For example, a song may accompany school or university years, or the early period with a partner. Sometimes there are songs we turn towards in difficult times to experience comfort and consolation. Is there a song that is related to specific phases of your life?
- Sometimes, we draw strength from **persons or material things that we’re not close** **to** personally, but that inspire us. For example, can you think of music from a movie that shaped your world view or music that you associate with a person you admire?

Search song version online on tablet computer or mobile phone (YouTube, Spotify,…)

- Is this the version of the song you imagined?

**Ending:**

Together, we have chosen the song ________ by ________. (Please document in protocol)

Does this feel right for you? Is there anything still on your heart you would like to say right now?

I would like to offer you to prepare the song for our next meeting and to play it for you in a slow and relaxed way. Would you be okay with that?

[Optional: You can ask the patient if they want a short music-based relaxation now to bring the first session to a close].

***Session 2: Live performance***

**How do I prepare?**

- At first, listen to the song several times and try to internalize it
- Pay attention whether the lyrics you’ve found correspond to the original song
- Play the chords from your sheets and compare it to the original song (the lyrics and sheets in the internet may not always be correct as harmonies are partially simplified)
- Try to make the lyrics and chords as similar as possible to the original song, especially at prominent passages

**Where do I find the song/sheets/lyrics?**

- In general: enter song title and “lyrics”, “chords” or “chords piano” in any search engine
- Different providers will appear that you can choose, e.g., [www.lyrics.com](http://www.lyrics.com) (lyrics), [www.ultimate-guitar.com](http://www.ultimate-guitar.com) (chords), [www.8notes.com](http://www.8notes.com) (piano sheets)
- You can search for music on YouTube or Spotify

**How do I approach the musical realization of the song?**

- At first, practice the song in the original style. Maybe adjust the key so you feel comfortable singing and you’re able to the sing the song with a calm and confident voice (if you use the guitar, you can use a capo)
- Then, you can either change the beat into a 3/4 or 6/8 beat. It should resemble the character of a lullaby, calm and peaceful.
- Tone intervals should be small, so change or simplify huge leaps or extreme passages
- Don’t play loudly, you can incorporate simple crescendos or decrescendos if adequate
- On the guitar, picking patterns should be preferred to strumming patterns
- Change, simplify or shorten difficult or critical phrases as the flow of music and easy understanding is the most important thing for the audience
- If the song has many verses or repetitions, shorten the song

**How do I begin the session?**

- Short welcome
- Hang a “Please don’t disturb” sign at the door
- Ask the patient to turn down the volume of their mobile phone
- Eliminate sources that may disturb the sessions (TV/radio, noise through open windows, etc.), if desired by the patient: ask their guests to leave the room for the duration of the session
- Position yourself with the instrument so you are turned towards the patient and are able to observe the breathing (torso/stomach)
- Position the music stand, maybe clip the pages so they won’t fall off
- Ask if the sitting or lying position is comfortable and if there can be made any improvements. Emphasize that it is important for the patient to be able to relax and feel good. Simultaneously add that the patient should not feel under pressure but to surrender to the situation as good as possible
- Shortly describe the procedure of the session: “At first, I will guide you in a short relaxation exercise and afterwards, I will play the song for you. When the song ends, I will leave the room for a few minutes so you can have a moment for yourself or to simply relax”

**How do I begin the short relaxation exercise?**

- Relaxation exercise completely on one chord (the first chord of the song)
- At first, play the chords several times and breathe calmly (as loud as the patient can hear it) and try to become aware if the patient seems relaxed and their breathing calm
- Speak quietly and calmly
- “Breathe in calmly and comfortably (wait until the patient breathed in) and breathe out (maybe join in breathing out)”
- “If possible, breathe out slightly longer than in, but only if it feels good to you”
- “If you want to, you can close your eyes” (if not already closed)
- Body scan: begin with the feet “Now, relax your feet…”, wait a few breaths, “…your calves…”, wait a few breaths, knees, thighs, hips, seat, lower back, belly, shoulders, chest, neck, jaw (here you can recognize if the patient is able to follow the relaxation when the jaw notably drops and the mouth slightly opens), forehead, skin of the head
- “The whole body becomes soft and sinks into the mattress. You can sense the bed holding you and you can completely let go”

**How do I transition to the Song of Life?**

- “In the next few minutes, as you listen to the sounds of your song, bring …. to mind” here you can name aspects of the background of the song, such as place, situation, persons etc. Imagine the situation yourself as well. Include as many senses and sensory impressions as possible in the creation of the image for the patient (e.g., smell the lavender fields, taste the salty water splashing into the boat, feel the warm sun on your skin…)
- Take a moment while the music continues to observe the breathing of the patient (chest or belly)
- Transition to the 3/4 or 6/8 beat as smooth as possible, maybe adjusted to the patient’s breathing. In 3/4 emphasize the first beat, in 6/8 both the first and the fourth. If the breathing is very fast, try to influence it through a slight decrease in tempo. Then, if applicable, play a short intro and sing the song.

**What do I do afterwards?**

- Depending on the situation leave the room (if the patient is very relaxed) or quietly inform the patient “I will leave the room now for about 5 minutes. You can continue to rest and give free rein to your feelings and thoughts”
- After 5 minutes, return to the patient
- Possible further procedure:
  - Gently enter the room and sit next to the patient in silence. Wait for the patient to begin to speak and respond to their statements
  - If the patient waits for the therapist to speak, you may ask “how are you feeling?”
  - Maybe make a few notes together with the patient what kind of important thoughts and feelings should be discussed in the third session or encourage the patient to make notes on their own

**What can/should I talk about?**

- As the reflection will take place in the third session, the second session should be completed with a short 5-minute conversation
- If the patient has a strong huge need to talk, be open for it, but make it short. Gently remind the patient of the third session in which those topics will be discussed
- Generally, validate outbursts of feelings and thoughts:
- “How beautiful that so many thoughts came up for you, they’re important”
- “You can draw on these beautiful memories anytime you’re sad or not feeling well. Remember the images and feelings well”
- “I am happy that you were able to relax. Remember the feeling so you’re able to go through this exercise alone with the CD”
- “Sadness also needs a place. Your need to cry was obviously important and necessary and I am happy that we created a safe space for your soul”
- Finally, shortly address the following session and the procedure. Set a time for the third session.

**How do I say goodbye?**

- “Tomorrow, we will see each other again and we can talk about your experience and feelings. I will bring a CD with the recording from today. I wish you all the best and I am looking forward to seeing you tomorrow”

***Session 3: Reflection (Interview guidelines)***

**Experience during the Song of Life (retrospective):**

- Spontaneous reaction:
  - When you think about the last session, what comes to your mind?
- Feelings:
  - Did specific memories, images, associations arise?
  - What feelings did you experience during listening?
  - Has something changed in your feelings (compared to before listening)? Did you experience new feelings?
- Thoughts:
  - What thoughts did you have during listening?
  - Has something changed in your thoughts (compared to before listening)?
- Physical sensations:
  - How did your body feel during listening?
  - Has something changed in your physical sensations (compared to before listening)? e.g., tension, muscles, breathing, heartbeat, specific regions of the body

**Effects:**

- Was participating in the music therapy rather positive/pleasurable/relaxing or negative/uncomfortable/burdening for you?
- What did it mean for you to listen to “your” song in this way?
- What does it mean for you that “your” song is captured on a CD?
- Have new perspectives opened up for you?
- When you take some time to process the situation associated with your Song of Life, do you feel like your view thereon has changed?
- Do you think that your view has changed with regard to other aspects and areas of your life, such as,
  - Things that are very important to you?
  - Things that you are proud of?
  - Things that you wish other people would remember about you?
  - Things that concern your current situation?

**Strengths:**

- What was particularly helpful for you in the three sessions of music therapy?

**Weaknesses:**

- What was difficult for you? / What was burdening for you?

**Conclusion:**

- Do you wish to give any other feedback to the study?
- Is there anything you need in order to feel good and complete? (maybe offer music-based relaxation)

1. **Control Group (Relaxation) (3 sessions)**

Structure for each session (20 minutes):

- 3 minutes introduction
- 12 minutes exercise
- 5 minutes debriefing

Introduction for the patient:

In the next few days, we will go through different relaxation exercises in three sessions. They each will last for about 20 minutes. In the beginning, I will tell you what the exercise will include, then we will practice together, and in the end we will talk about your experience during the exercise. The aim of the exercises is to contribute to relaxation and inner calm, as far as possible in your current situation.

***Session 1: progressive muscle relaxation (PMR)***

Today, we will practice becoming aware of the change between tension and relaxation. In the next session, we will focus on the breath, and the final session will involve an imaginary journey.

**Introduction:**

The aim of today’s exercise is to consciously perceive the difference between tension and relaxation. This may support you in feeling better no matter the life situation. I will now guide you step by step through the exercise. Do you have any questions before we begin? [Note: adapt the exercise depending on the general state of the patient to only slight or imaginary tension and relaxation]

**Exercise:**

Let’s begin with the exercise.

Body posture: Turn your attention inwards for a moment. Sense your contact with the bed. Are you lying comfortably? Or do you wish to change something in your position so you can rest as comfortably as possible for the next ten minutes (pillows, rolls, height adjustment…)?

If it’s comfortable for you, close your eyes. If not, let your eyes be half open.

Observe how sounds come and go.

Observe how feelings and thoughts come and go.

Focus your attention on your feet and legs: Push your heels down and away as best as you can and tense the leg muscles. Hold the tension and continue to breathe (2-3 breaths). Now release the tension. Pay attention to the difference between the tension before and the relaxation now. Stay with your focus on feet and legs (2-3 breaths). Now repeat it as well as possible: Push both heels down and away as best as possible and tense the leg muscles. Hold the tension and continue to breathe (2-3 breaths). Now release the tension. Pay attention to the difference between the tension before and the relaxation now. Stay with your focus on both feet and legs (2-3 breaths).

Focus your attention on your pelvis and bottom: Tense the gluteal muscles and push your pelvis up as well as possible. Hold the tension and continue to breathe (2-3 breaths). Now release the tension. Pay attention to the difference between the tension before and the relaxation now. Stay with your focus on your pelvis and bottom (2-3 breaths). Now repeat: Tense the gluteal muscles and push your pelvis up as best as you can. Hold the tension and continue to breathe (2-3 breaths). Now release the tension. Pay attention to the difference between the tension before and the relaxation now. Stay with your focus on your pelvis and bottom (2-3 breaths).

Focus your attention on your upper body and chest: Try to open and expand your chest and contract the shoulder blades, as best as you can. Hold the tension and continue to breathe (2-3 breaths). Now release the tension. Pay attention to the difference between the tension before and the relaxation now. Stay with your focus on your upper body and chest (2-3 breaths). Now repeat: Try to open and expand your chest and contract the shoulder blades, as well as possible. Hold the tension and continue to breathe (2-3 breaths). Now release the tension. Pay attention to the difference between the tension before and the relaxation now. Stay with your focus on your upper body and chest (2-3 breaths).

Focus your attention on your hands and arms: Clench both hands into fists with the thumb inwards, as best as you can. Hold the tension and continue to breathe (2-3 breaths). Now release the tension. Pay attention to the difference between the tension before and the relaxation now. Stay with your focus on your hands and arms (2-3 breaths). Now repeat: Clench both hands into fists again with the thumb inwards, as well as possible. Hold the tension and continue to breathe (2-3 breaths). Now release the tension. Pay attention to the difference between the tension before and the relaxation now. Stay with your focus on your hands and arms (2-3 breaths).

Stay with your attention on your hands and arms: Open up your hands as well as possible and stretch all fingers. Hold the tension and continue to breathe (2-3 breaths). Now release the tension. Pay attention to the difference between the tension before and the relaxation now. Stay with your focus on your hands and arms (2-3 breaths). Now repeat: Open up your hands again as well as possible and stretch all fingers. Hold the tension and continue to breathe (2-3 breaths). Now release the tension. Pay attention to the difference between the tension before and the relaxation now. Stay with your focus on your hands and arms (2-3 breaths).

Focus your attention on your head and face: Try to tense your face: frown the forehead, narrow the eyes, wrinkle your nose, tense your cheeks, press your lips together, clench your jaw and teeth, tense the whole scalp, as best as you can. Hold the tension and continue to breathe (2-3 breaths). Now release the tension. Pay attention to the difference between the tension before and the relaxation now. Stay with your focus on your head and face (2-3 breaths). Now repeat and tense your face: frown the forehead, narrow the eyes, wrinkle your nose, tense your cheeks, press your lips together, clench your jaw and teeth, tense the whole scalp, as best as you can. Hold the tension and continue to breathe (2-3 breaths). Now release the tension. Pay attention to the difference between the tension before and the relaxation now. Stay with your focus on your head and face (2-3 breaths).

Now, feel the relaxation from head to toe. Feel your body as a whole. Let the relaxation deepen with every exhale. Sense the calm in every part of your body.

We will now come to a close. If you want, count to three, and come back with your attention into the room and open your eyes.

**Debriefing:**

What did you experience during the exercise – on the level of the body, feelings and thoughts? Were there any difficulties, blocks or obstacles? If so, where were they? Were you able to let go into and sense the relaxation? If so, where was it the most obvious? As you transition into the day and the week, you may remember the relaxation and calm. Even if you don’t practice directly, it may bring some relief.

***Session 2: Focus on the breath***

**Introduction:**

The aim of today’s exercise is to consciously become aware of the process of breathing – breathing in and out and the breaks in between – and to align with the present moment. It’s about an inner attitude to surrender to the breath just like it’s happening without trying to influence or change it. This may support you in feeling better regardless of the situation you find yourself in. I will now guide you step by step through the exercise. Do you have any questions before we begin?

**Exercise:**

Let’s begin with the exercise.

Body posture: Turn your attention inward for a moment. Sense your contact with the support. Are you lying comfortably? Or do you wish to change something in your position so you can rest as comfortably as possible for the next ten minutes (pillows, rolls, height adjustment…)?

If it’s comfortable for you, close your eyes. If not, let your eyes be half open.

Observe how sounds come and go.

Observe how feelings and thoughts come and go.

- Focus your attention gently on your breath.
- Stay with your attention where you can feel the breath the most (nose, belly).
- Concentrate on the sensations that emerge with breathing.
- Become aware of the whole length of breathing in and out as well as the breaks between the breaths. Breath by breath.
- Surrender the breath to itself, there is nothing for you to do, breathing happens on its own.
- Sometimes you may want the breath to be different and to change it – this is a natural tendency, just recognize it. Then let go of the tendency to control the breath and focus your attention on how the breath comes in and out. Stay with this perception.
- Maybe thoughts, feelings, physical sensations, sounds or daydreams arise and you recognize yourself thinking about something… if this happens, simply take note of it without judgment. This is a natural process: Thoughts come and go. And you can learn to become aware of it.
- Come back gently to the attention on the breath.
- Now, slowly come back to the awareness of your body. Feel your body in its posture and contact to the support.
- Expand your awareness into the room. Very slowly come back to the room and open your eyes
- If an impulse arises to move, follow it.

**Debriefing:**

What did you experience during the exercise – on the level of the body, feelings and thoughts? Were there any difficulties, blocks or obstacles? If so, where were they? Were you able to surrender to the perception of the breath? If so, where was it most obvious? Were there distractions and was it possible for your attention to come back to your breath? As you transition into the day and the week, you may remember your breath and for a moment feel the flow of the breath here and now.

***Session 3: Imaginary journey***

**Introduction:**

The aim of today’s exercise is to find an imaginary inner place where you feel safe and relaxed. This may support you in feeling better regardless of the situation you find yourself in. I will now guide you through the exercise step by step. Do you have any questions before we begin?

**Exercise:**

Let’s begin with the exercise.

Body posture: Focus your attention inward for a moment. Sense your contact with the support. Are you lying comfortably? Or do you wish to change something in your position so you can rest as comfortably as possible for the next ten minutes (pillows, rolls, height adjustment…)?

If it’s comfortable for you, close your eyes. If not, let your eyes be half open.

Observe how sounds come and go.

Observe how feelings and thoughts come and go.

You can always come back to your breath to find your way back to the here and now.

In the following, I would like to invite you to imagine being at a place in nature which conveys calm and relaxation. You can use the associations and images which naturally arise in you.

It may be a place you know, where you have been personally. Or it may be a place in your imagination or a place you have seen or heard about in a book or a movie. Let an inner space arise, a space in nature: in the forest, in the mountains, on a meadow, at the sea. Observe what arises in you and stay with one of these natural environments.

Now be at that place with all your senses. Let your eyes wander: what do you see? Colors, forms, light… Are there sounds at that place? Maybe birds singing, wind in the trees, rain drops… Is there a particular smell? How does it feel to be at that specific place? What do you sense?
It may be that one of the senses is in the foreground. The place might appear clear or unclear for your inner eye. Just as it is right now it is perfect.

To be at this place right now, what is it like for you? How does it feel? Which physical sensations can you observe? Are there any thoughts? Or feelings?

Receive the atmosphere of this place, become aware of the connection with yourself and this place and breathe.

Very slowly, say goodbye to this place. For a last time, feel the place consciously.

Now come back slowly to the awareness of your body. Feel your body in its position and contact to the support. Expand your awareness into the room. Very slowly come back to the room and open your eyes. If an impulse arises to move, follow it.

**Debriefing:**

What did you experience during the exercise – on the level of the body, feelings and thoughts? Were there any difficulties, blocks or obstacles? If so, where were they? Were you able to surrender to the imagination of this place? If so, in which way? As you transition into the day and the week, you may remember this place and receive the calm and relaxation of it.
